# Supplementary material for: Graphene Microelectrode Arrays, 4D Structured Illumination Microscopy, and a Machine Learning Spike Sorting Algorithm Permit the Analysis of Ultrastructural Neuronal Changes During Neuronal Signaling in a Model of Niemann–Pick Disease Type C
Source: Adv Sci (Weinh). 2024 Sep 28;11(44):2402967. doi: 10.1002/advs.202402967 (PMC11600250; doi:10.1002/advs.202402967)
Supplement: Supplementary file 1 — Supporting Information [file ADVS-11-2402967-s003.docx]

**Supplementary Information**

**Graphene Microelectrode Arrays, 4D Structured Illumination Microscopy, and a Machine Learning Spike Sorting Algorithm Permit the Analysis of Ultrastructural Neuronal Changes During Neuronal Signalling in a Model of Niemann-Pick Disease Type C**

**Meng Lu*, Ernestine Hui*, Marius Brockhoff***, Jakob Träuble, Ana Fernandez-Villegas, Oliver J Burton, Jacob Lamb, Edward Ward, Philippa J Woodhams, Wadood Tadbier, Nino F Läubli, Stephan Hofmann, Clemens F Kaminski, **Antonio Lombardo^†^, Gabriele S Kaminski Schierle^†^**

*These authors contributed equally to this work.

^†^Corresponding author email address:

gsk20@cam.ac.uk (G.S.K.S), a.lombardo@ucl.ac.uk (A.L.)

**Table of Contents**

**Information S1.** Large-scale synthetic dataset for development and benchmarking of the spike sorting algorithm 2

**Table S1.** Overview number of spikes per class for the Small dataset 2

**Table S2.** Overview number of spikes per class for the Large dataset. 3

**Table S3.** Benchmarking performance (accuracy) of several ML spike sorting approaches 3

**Figure S1.** Signal-to-noise ratio comparison of raw data recorded using graphene MEAs and ITO MEAs. 4

**Figure S2.** Correlated local electrophysiological and calcium activity recordings from different locations on G-MEAs highlight variations in signals including reduced correlation between events of the signal types as well as differences in firing rates. 5

**Figure S3.** Single neuron structure and calcium activity. 6

**Movie S1.** A representative neuronal structure is imaged using sectioning SIM in a time-lapse sequence to illustrate changes in structure and calcium activity. 6

**Movie S2:** A representative neuronal network is imaged using sectioning SIM in a time-lapse sequence to illustrate changes in structure and calcium activity. 6

**Movie S3:** A representative neuronal structure at control condition is imaged using sectioning SIM in a time-lapse sequence to illustrate changes in structure and calcium activity. 6

**Movie S4:** A representative neuronal structure treated with U18666A is imaged using sectioning SIM in a time-lapse sequence to illustrate changes in structure and calcium activity. 6

**Acronyms** 7

**Units** 7

**Information S1. Large-scale synthetic dataset for development and benchmarking of the spike sorting algorithm**

The presented datasets aim to provide an extensive, ground-truth basis for the evaluation and benchmarking of spike-sorting algorithms. Unfortunately, currently available datasets are too simple and small to realistically allow to capture the recent, mostly machine-learning-driven, advances in the field or to reflect the magnitude of spikes recorded by the latest generation of MEAs. The datasets contain simulated spike shape recordings generated *via* NeuroCube^[40]^. To create the recordings, the default settings of NeuroCube have been used (single electrode, 300.000 neurons/mm^3^, 7 % rate of active neurons, exponential firing rates). Recordings were simulated with a sampling frequency of 20 KHz. For each recording, five neurons (maximum available) have been manually placed around a single recording electrode. The relative distance to the electrode has been randomly sampled between 0 and 1 (step size 0.01) and firing rates have been randomly sampled between 15 and 35 Hz. Alongside the datasets, we provide the created Cube files that can be loaded into the NeuroCube software in order to simulate the same conditions and neurons that have been used here.


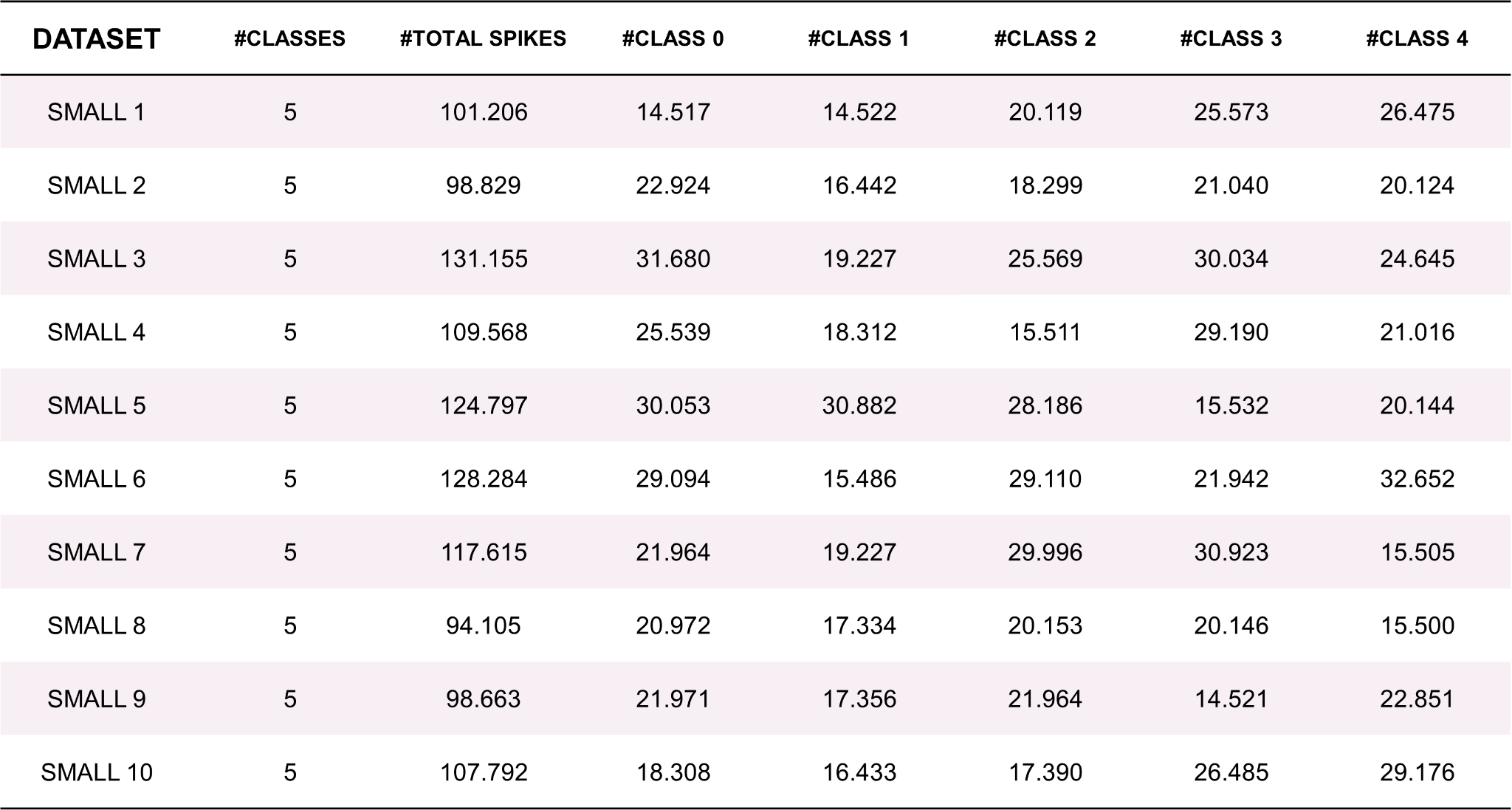


**Table S1.** Overview number of spikes per class for the *Small* dataset.

Data is stored as Python pickle files. Each pickle file can be loaded with a simple pickle.load() command. Stored is an array of dimensions Number_of_spikes x 66. The first column carries the ground-truth spike class for each spike (integer). The second column contains the spike time of the simulated spike (in ms). Finally, columns 3 to 66 contain the respective spike shape (64 data points).

The datasets are organised by size and complexity. The group of sets called *Small* are 10 sets of spike recordings, each including about 100,000 spike shape recordings of 5 classes (source neurons). The spike shapes and firing rates present in each set have been chosen randomly. Details on spike shapes as well as the number of recordings of each neuron can be found in Supplementary Table 1.

The datasets called *Large* are 10 sets of spike recordings, each including about 1,100,000 spike shape recordings of 5 classes (source neurons). The spike shapes and firing rates present in each set have been chosen randomly. Details on spike shapes as well as the number of recordings of each neuron can be found in SI Table 2.

**
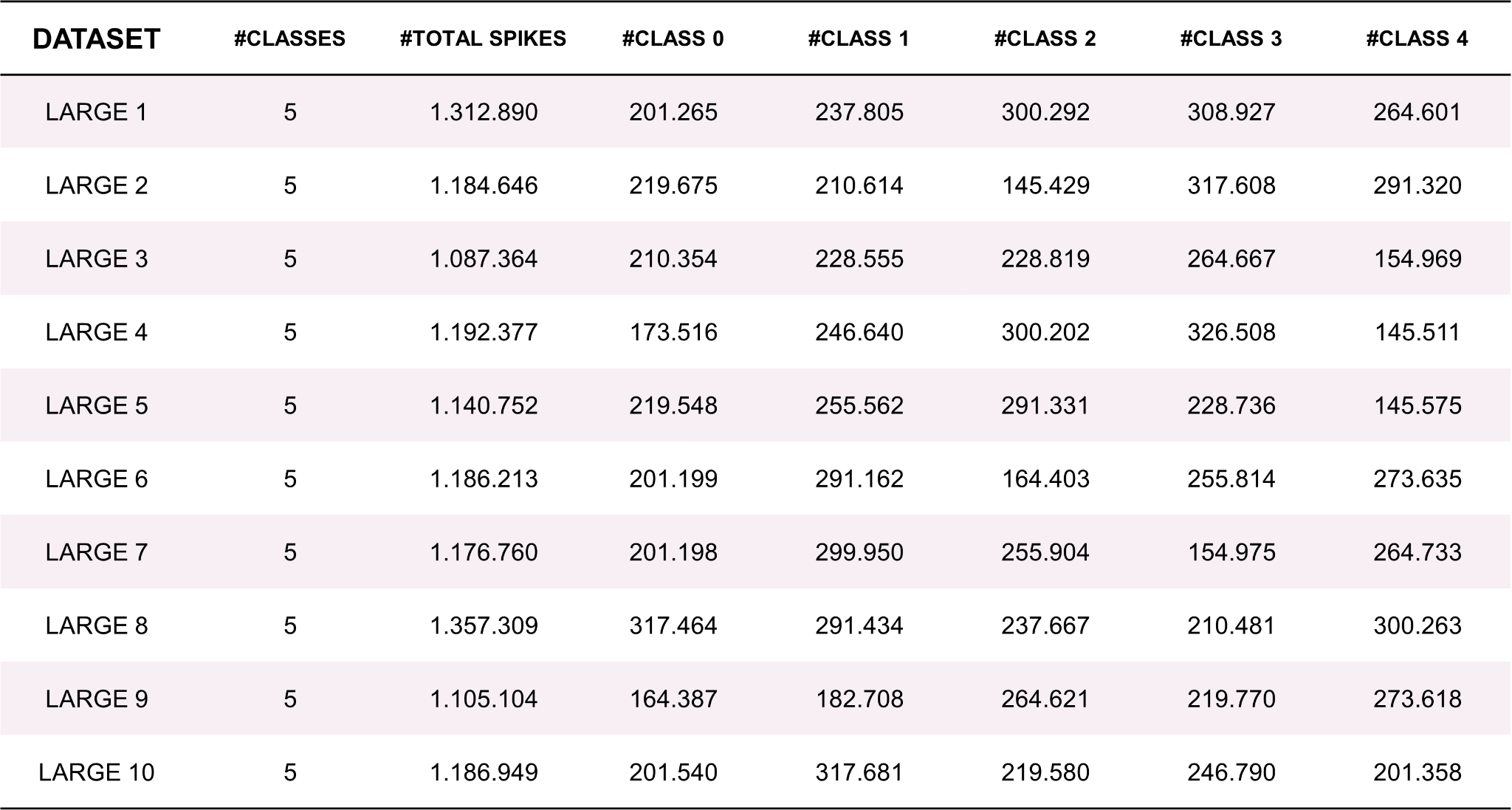
**

**Table S2.** Overview number of spikes per class for the *Large* dataset.

**Benchmarking deep clustering algorithms on large-scale synthetic dataset**


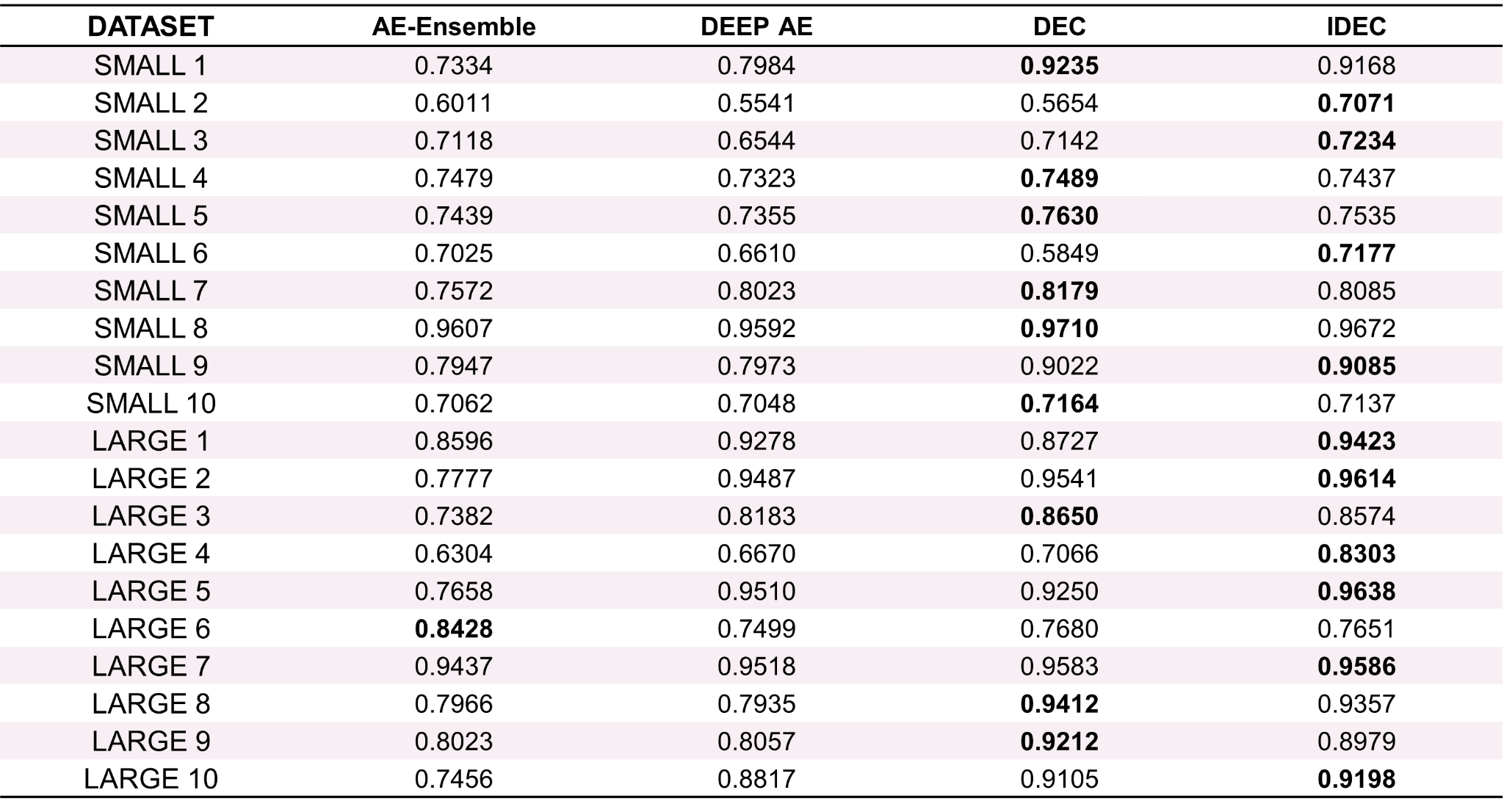


**Table S3.** Benchmarking performance (accuracy) of several ML spike sorting approaches. Given is the best result of 5 repeated runs. To enable a fair comparison, we compare accuracies obtained *via* K-means++^[70]^ clustering with the correctly given number of clusters. Best performing model per dataset is marked in bold.


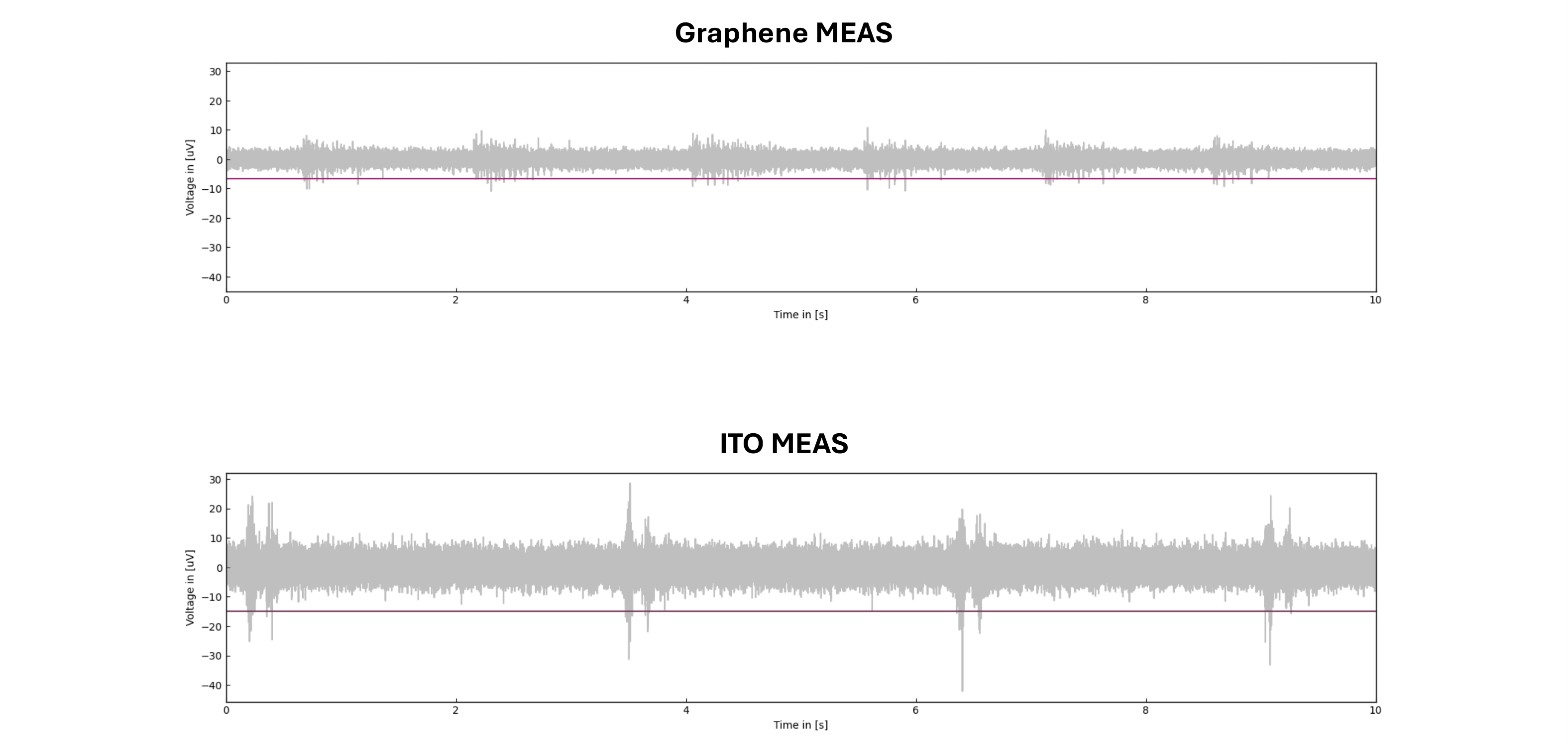


**Figure S1.** Signal to noise ratio comparison of raw data recorded using graphene MEAs and ITO MEAs.


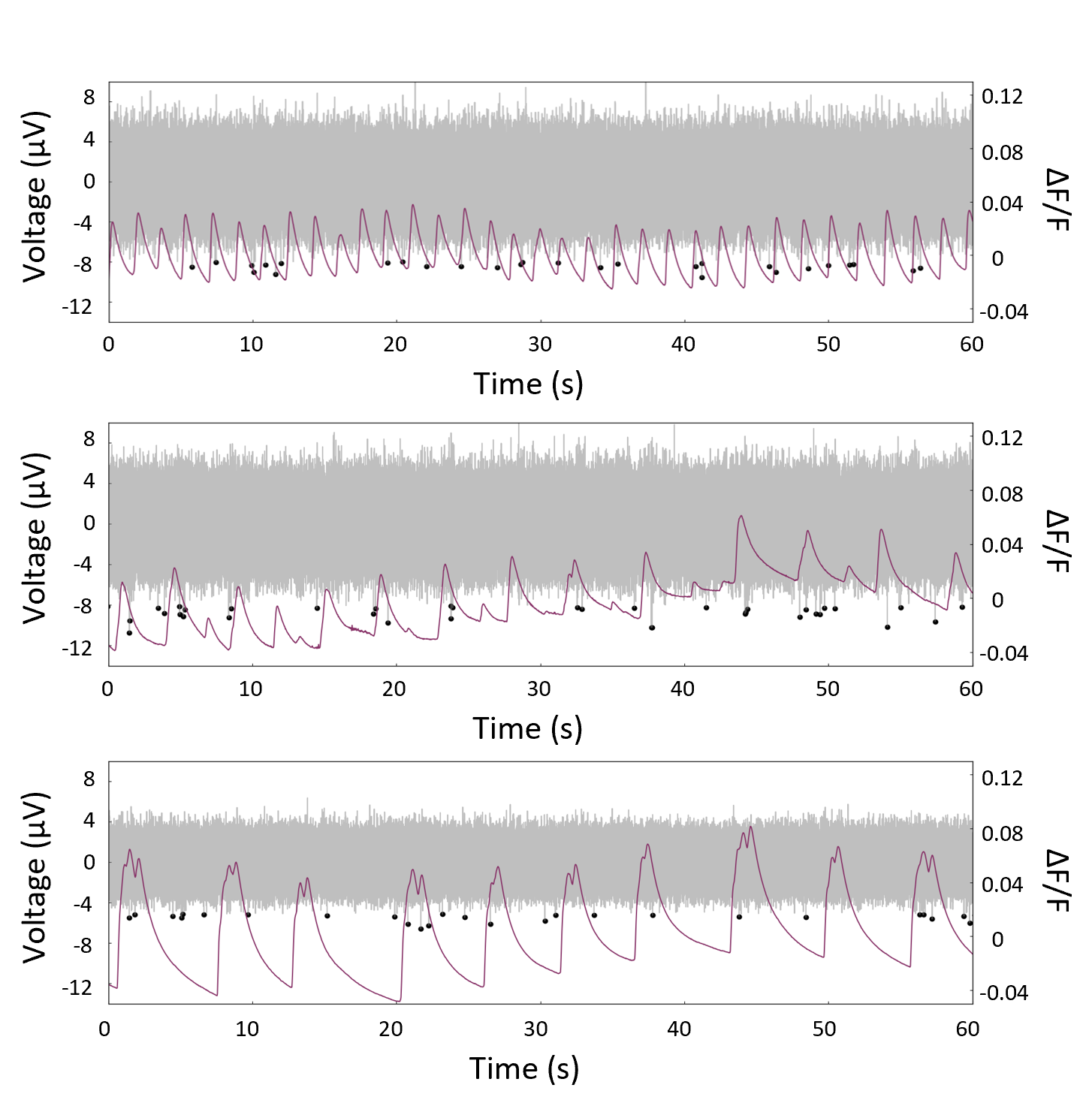
.

**Figure S2.** Correlated local electrophysiological and calcium activity recordings from different locations on G-MEAs highlight variations in signals including reduced correlation between events of the signal types as well as differences in firing rates.


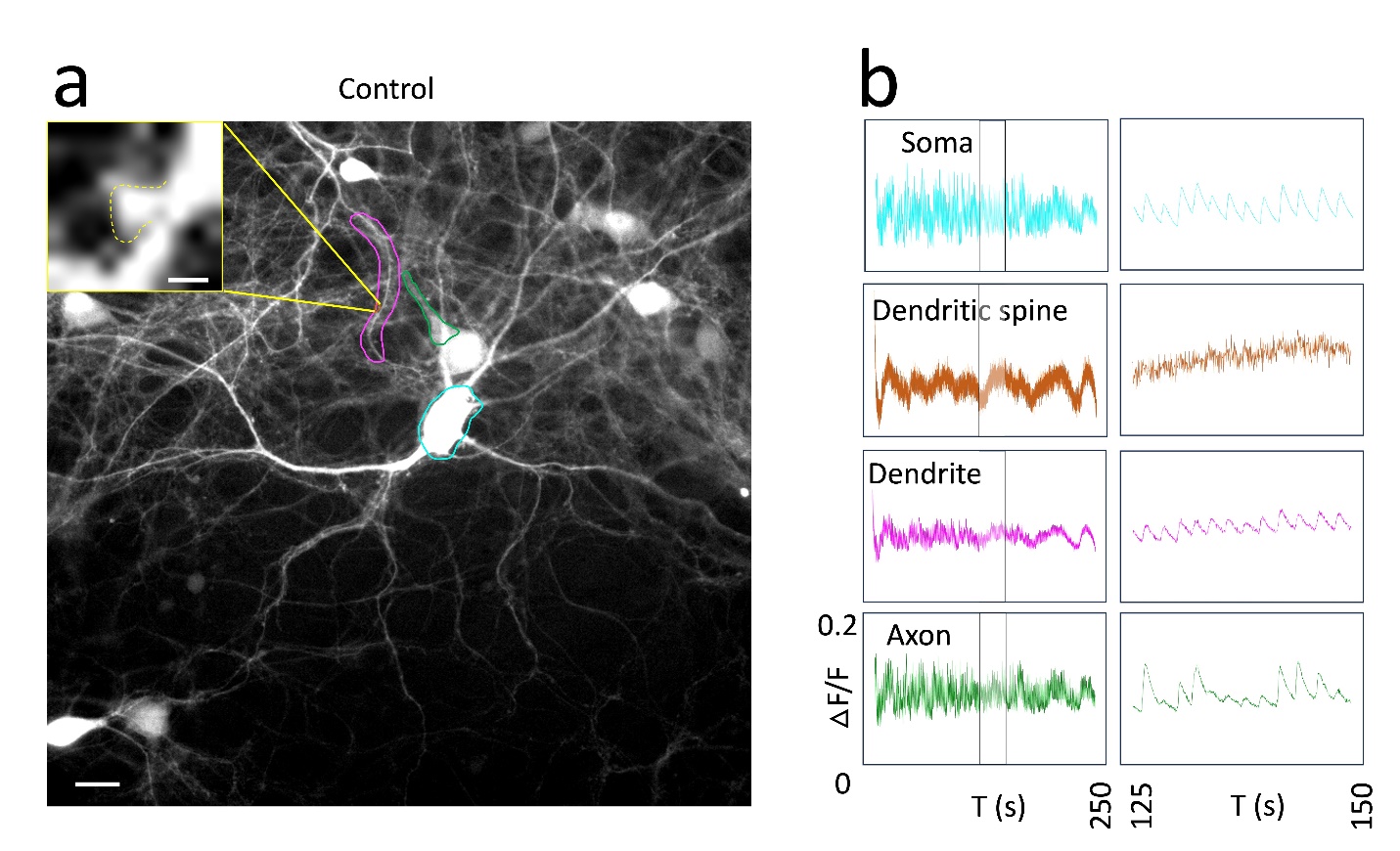


**Figure S3.** Single neuron structure and calcium activity. (**a**) The structure of a single neuron in a single FOV. Sub-neuronal structures highlighted in the figure are a dendrite (magenta), an axon (green), a soma (cyan), and a dendritic spine (brown). The enlarged boxed region highlights the dendritic spine analysed in b. Scale bars: 20 µm, 1 µm for enlarged FOVs. Individual images were acquired by 10 ms exposure time with a laser emitting at a wavelength of 488 nm, 40x magnification with a lens length extender. Four independent experiments were performed. (**b**) Changes in the amplitude of the calcium sensor fluorescence extracted from the neuronal structures are depicted over a time window of 250 s. Y-axis: ΔF/F with a scale of 0 to 0.6 for all the plots. The small time-window (25 s, right side) is indicated by black rectangles in the full-time window panel (left side). Sub-plots have the same y-axis and scales.

**Movie S1:** A representative neuronal structure is imaged using sectioning SIM in a time-lapse sequence to illustrate changes in structure and calcium activity. The movie was recorded over a time frame of 40 s. Calcium spikes can be clearly visually detected in the recording, accompanying changes of sub-neuronal structures such as single spines. 20 time points and 15 sections for each timepoint were imaged.

**Movie S2:** A representative neuronal network is imaged using sectioning SIM in a time-lapse sequence to illustrate changes in structure and calcium activity. The movie was recorded over a time frame of 24 s. 18 time points and 10 sections for each timepoint were imaged. The axons and dendrites intricately interweave, forming a dense mesh that signifies a high degree of interconnectivity crucial for efficient signal transmission and neural plasticity.

**Movie S3:** A representative neuronal structure at control condition is imaged using sectioning SIM in a time-lapse sequence to illustrate changes in structure and calcium activity. The movie was recorded over a time frame of 40 s, showing the normal size of synaptic boutons. 20 time points and 12 sections for each timepoint were imaged.

**Movie S4:** A representative neuronal structure treated with U18666A is imaged using sectioning SIM in a time-lapse sequence to illustrate changes in structure and calcium activity. The movie was recorded over a time frame of 40 s, showing a significant increase in size of synaptic boutons. 20 time points and 12 sections for each timepoint were imaged.

**Acronyms**

Graphene microelectrode arrays (G-MEAs)

Niemann-Pick disease type C (NPC)

Microelectrode arrays (MEAs)

Field of view (FOV)

Indium tin oxide (ITO)

Machine Learning (ML)

Structured illumination microscopy (SIM)

Deep Embedding for Clustering (DEC)

Improved Deep Embedding for Clustering (IDEC)

Autoencoder-Ensemble (AE-Ensemble)

Standard deep autoencoder (Deep AE)

Day *in vitro* (DIV)

Adeno-associated viruses (AAV)

Four-dimensional structured illumination microscopy (4D-SIM)

Three-dimensional (3D)

Fluorescence lifetime imaging microscopy (FLIM)

Fluorescence resonance energy transfer (FRET)

**Units**

Ohms (Ω)

micrometres (𝜇m)

reciprocal centimetre (cm^-1^)

millimetres squared (mm^2^)

kilo Hertz (kHz)
